# Estimation of pollen dispersal distance in Job’s tears (*Coix lacryma-jobi* L.) by using red leaf sheath as a morphological marker

**DOI:** 10.1270/jsbbs.23016

**Published:** 2023-09-09

**Authors:** Katsuhiro Matsui, Takayuki Tamura, Keito Nishizawa, Akiko Ohara-Takada

**Affiliations:** 1 Institute of Crop Science, National Agriculture and Food Research Organization (NARO), Kannondai 2-1-2 Tsukuba, Ibaraki 305-8518, Japan; 2 Institute of Life and Environmental Sciences, University of Tsukuba, Kannondai 2-1-2 Tsukuba, Ibaraki 305-8518, Japan; 3 Center for Medicinal Plant Resources, Toyama Prefectural Institute for Pharmaceutical Research, 2732 Hirono, Kamiichi-machi, Nakaniikawa, Toyama 930-0412, Japan; 4 Institute of Vegetable and Floriculture Science, NARO, 360 Kusawa, Ano-cho, Tsu, Mie 514-2392, Japan; 5 Research Center of Genetic Resources, NARO, Kannondai 2-1-2 Tsukuba, Ibaraki 305-8602, Japan

**Keywords:** Job’s tears, morphological marker, outcrossing, wind pollination, regression analysis

## Abstract

Job’s tears (*Coix lacryma-jobi* L.) is grown widely in Asian countries and a crop that can fertilize with own pollen and pistils. The grains are used not only for food but also for medicinal purposes. The grain of many cultivars contains glutinous endosperm; only grains with this glutinous endosperm are suitable for use as medicine in Japan. Many wild types have non-glutinous endosperm and can easily cross with cultivar under natural environmental conditions. Because the non-glutinous endosperm trait is dominant to that of glutinous endosperm, F_1_ seeds produced by crosses between a cultivar and a wild type have non-glutinous endosperm. To reduce the rate of unwanted crosses, we investigated the pollen dispersal distance by using a red leaf sheath as a morphological marker. When plants were cultivated in rows 70 cm apart, the crossing rate was about 25%–35%. As the distance increased, the crossing rate decreased at a rate that could be fitted to a power approximation in fields without intervening plants and to an exponential equation in fields with intervening plants. Our data could be used as guidelines for preventing unwanted crossing with wild types when growing cultivars.

## Introduction

Job’s tears (*Coix lacryma-jobi* L.) is widely grown in Asian countries, such as China, Korea, India, Indonesia, and Japan, and the grains are used medicinally as well as for food (Arora 1977). Plants of cultivars, such as *C. lacryma-jobi* var. *ma-yuen*, have a soft hull, and in most cultivars the grain has glutinous (waxy) endosperm (Hachiken *et al.* 2012, Kempton 1921). Only grains with this glutinous endosperm are suitable for use as medicine in Japan, as stated in the Japanese Pharmacopoeia (Ministry of Health, Labour and Welfare 2016; https://www.mhlw.go.jp/stf/seisakunitsuite/bunya/0000066597.html).

Plants of the wild type, such as *C. lacryma-jobi* var. *lacryma-jobi* have a hard seed hull, and the wild type has a lot of diversifications controlled genetically (Xi *et al.* 2016). The wild type grows in large areas across Japan, preferentially in warm wet habitats such as near rivers and ponds. Grains of the wild type usually have non-glutinous (non-waxy) endosperm (Hachiken *et al.* 2012, Kempton 1921).

Both types are plants which can fertilize with own pollen and pistils, but because the flowers have protruding stamens and pistil (Supplemental Fig. 1), plants do not obligately self-pollinate and can easily outcross, even among different types. From this reason, production of a cultivar can sometimes become contaminated with non-glutinous endosperm (Okuyama *et al.* 1989), and in fact some landraces, such as Okayama-zairai, are recognized to have a certain ratio of non-glutinous endosperm (Hachiken *et al.* 2012, Okuyama *et al.* 1989). In addition, the trait of non-glutinous endosperm is dominant to glutinous endosperm and shows a xenia effect, in which pollen affects the characteristics of seeds of the fertilized plant. Therefore, the F_1_ seeds produced by a cross between cultivated and wild types have non-glutinous endosperm, even if those seeds are on the plants of a glutinous cultivar. For this reason, it is important to ensure that plants used for medicinal purposes in Japan are not crossed with wild non-glutinous types. However, the rate of outcrossing and the pollen dispersal distances under cultivation conditions are not known.

Qualitative traits have been used as morphological markers for genetic analysis and selection in many crops. A qualitative trait that is controlled by a single gene, or by a few genes, is especially well suited to this use. Color traits caused by the accumulation of pigments are one useful type of marker. The leaf sheath color of Job’s tears can be either green or red (including pale red and purple), and the colors can be easily distinguished in the field.

Here, we investigated the probability of crossing with other population under cultivation conditions and estimated the pollen dispersal distance by using a red-sheath morphological marker in Job’s tears. We also discuss the implications for cultivation methods in areas where the wild type is thought to be present.

## Materials and Methods

### Plant materials

**Fig. 1.**
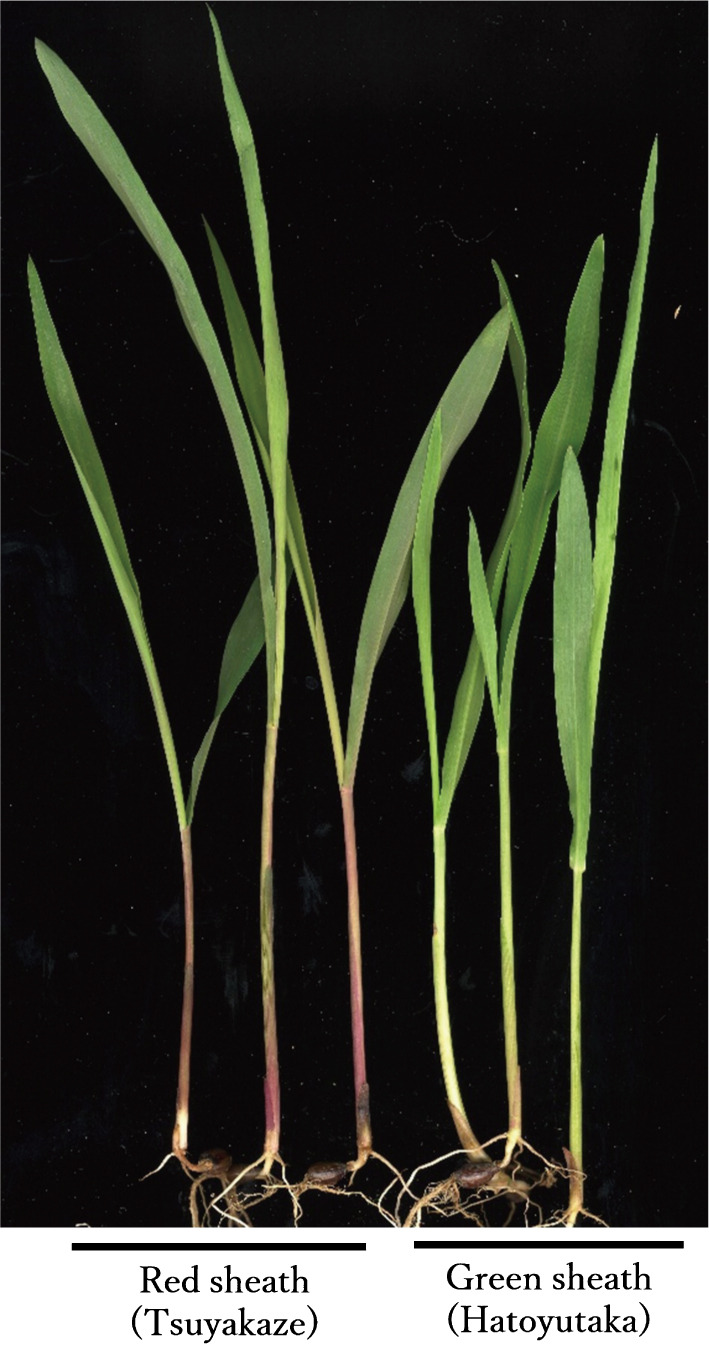
Sheath color of seedlings of Job’s tears. The left three plants are ‘Tsuyakaze’, with red leaf sheaths, and the right three plants are ‘Hatoyutaka’, with green leaf sheaths.

There are two main sheath colors, red and green, in common cultivars of Job’s tears in Japan (Hara *et al.* 2007; Fig. 1). Three red-sheath cultivars, ‘Akishizuku’ (AKI; Tetsuka *et al.* 2010), ‘Toriizumi’ (TRI; Tetsuka *et al.* 2014), and ‘Tsuyakaze’ (TUY), and two green-sheath cultivars or breeding lines, ‘Hatoyutaka’ (HAT; Kato *et al.* 2007) and gs-TUY, which was developed by selection from a TUY breeding population, were used for the pollen dispersal test. Genetic analysis of the sheath color trait was performed with TUY and HAT. F_1_ plants were produced by a cross between HAT and TUY. Three F_2_ segregating lines were produced by self-pollination of the F_1_ plants.

### Estimation of pollen dispersal distance

We conducted the following four experiments: Experiment 1, Estimation of crossing rate under close cultivation; Experiment 2, Estimation of pollen dispersal distance by planting at regular intervals; Experiment 3, Estimation of pollen intrusion distance into a cultivar from bordering plants of a different cultivar; and Experiment 4, Estimation of pollen dispersal rate into a cultivar from plants of a different cultivar on the basis of unit acreage sampling. All experiments were performed in 2021 and 2022.

**Fig. 2.**
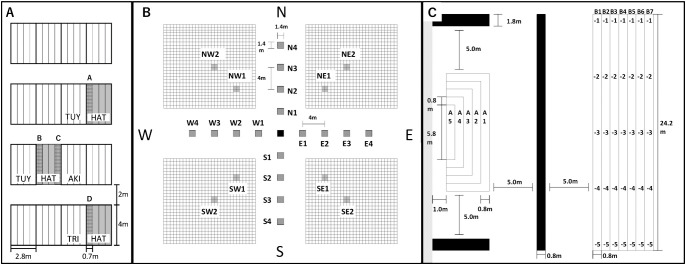
Planting design for each experiment. A, Planting design used to investigate crossing rates under closely planted conditions. Red-sheath plants were cultivated in areas shown in white, and green-sheath plants were cultivated in areas shown in grey. Seeds were collected from plants in the mesh area to investigate the crossing rate. Tsuyakaze (TUY), Akishizuku (AKI), and Toriizumi (TRI) were cultivated next to the green-sheath plants. B, Planting design used to investigate pollen dispersal distance. In the center area (black square), Tsuyakaze (red sheath) was cultivated. The areas marked with grey squares and cardinal directions (N, E, S, W) were planted with Hatoyutaka (green sheath) in 2021 and gs-TUY (green sheath) in 2022. Hatoyutaka was planted in the mesh area in 2022. C, Planting design used to investigate pollen intrusion distance. In the black areas, Akishizuku (AKI, red sheath) was cultivated and HAT plants were cultivated in areas A and B. In the area on the left (A1–A5), plants were harvested from the edge, at 0.8-m intervals. In the area on the right (B), plants were harvested from five areas in each row, at 0.8-m intervals. In the area shown in gray on the left, peony plants about 60 cm tall were growing in 2021.

Experiments 1 and 2 were performed in two different fields, about 10 km apart, at the Institute of Crop Science, National Agriculture and Food Research Organization (NARO), Japan. Maps of the planting schemes for each experiment are shown in Fig. 2.

In Experiment 1, plants were grown in 4-m-long rows (*n* = 4 in an experimental block) with 25 plants in a row that were 0.7 m apart (Fig. 2A). HAT (green-sheath) plants were placed next to rows of TUY, AKI, and TRI (red-sheath) plants. Four areas (A to D) were harvested and the seeds were grown to determine whether they would produce red or green leaf sheaths. TUY, AKI, and TRI were expected to be the main sources of red-sheath pollen. The crossing rate was estimated by using more than 100 seeds harvested from all plants in the row.

In Experiment 2, red-sheath TUY plants were grown in the center of the plot, and green-sheath HAT or gs-TUY plants were grown on the edges of the plot. In 2021, four TUY plants were planted at 0.7-m distance in the center area, and four plants of HAT (green, maternal line) were planted in each of four areas radiating out from the center in the four cardinal directions (N, E, S, W), at 4-m intervals. Furthermore, two additional areas (two 40-m long rows separated by 0.7 m), about 100 m to the SE (YB7) and NW (YA5) from the center TUY were planted with HAT (not shown on the map of Fig. 2B).

In 2022, the green-sheath line gs-TUY was planted instead of HAT to reduce the effect of genetic background. In addition, nine TUY plants were planted in the center, at 35 cm distance, to increase the pollen source. HAT was planted in the empty areas marked with mesh squares in Fig. 2B, and two additional areas to each of the SE, SW, NE, and NW were set as shown in Fig. 2B.

Experiments 3 and 4 were performed in a field at the Center for Medicinal Plant Resources, Toyama Prefectural Institute for Pharmaceutical Research, by using red-sheath AKI and green-sheath HAT plants. The experimental design is shown in Fig. 2C. Large areas (4 m × 12.2 m) of HAT plants at 0.8 m distance were grown, bordered by long rows of AKI plants with the distance 5.0 m from HAT. In Experiment 3, seeds of plants were harvested from the edge of the large area at 0.8 m increasing distance areas (A1–A5). In Experiment 4, HAT plants were grown on a large 5.6 m × 24.2 m plot with 7 rows at 0.8 m distance, and a long row of AKI 5.0 m away from the HAT was created in front of it. Four small areas (0.8 m × 1.0 m) were harvested in a row. The rate of crosses between cultivars was estimated by determining the ratio of red to green sheath in plants grown from seeds harvested in each area.

## Results

### Analysis of genetic dominance and inheritance of red-sheath trait

To evaluate whether red sheath could be used as a morphological marker, we investigated the dominance of the color in F_1_ seedlings produced by a cross between the green-sheath cultivar ‘Hatoyutaka’ (HAT) and the red-sheath cultivar ‘Tsuyakaze’ (TUY). We obtained 12 seeds from the cross, and they produced 11 F_1_ seedlings with red sheaths and one with a green sheath. The red sheath thus appears to be dominant over green, and the one green seedling was likely produced by self-pollination. To confirm dominance and estimate the number of genes controlling sheath color, we developed three segregating lines (Seg_1, Seg_2, and Seg_3) derived from three individual F_1_ plants from HAT × TUY crosses and investigated the segregation of sheath color.

In the three F_2_ lines, sheath color followed the 9:7 ratio expected for a trait controlled by two complementary genes (Table 1). Although it is not clear which pigments produce the red sheath, at least two genes may be involved in the pigment synthesis pathways and may lose these functions in HAT

### Crossing rate of Job’s tears under common cultivation conditions

We investigated the crossing rate by sampling four areas where green-sheath HAT and red-sheath TUY plants were cultivated in rows 70 cm apart (Fig. 2A). Crossing rates ranged from 14.7%–29.8% in 2020 and 16.1%–35.4% in 2021 (Table 2).

### Estimation of pollen dispersal distance from crossing rate of plants grown at equal intervals

To estimate the regularity of pollen dispersal, we investigated the crossing rates of green-sheath plants grown at 4-m intervals from red-sheath plants to the north, south, east, and west (Fig. 2B). The crossing rate varied among areas, depending on the direction and the year. The crossing rates of plants to the east and west were lower than those of plants to the north and south in 2021 and 2022. The highest crossing rate was observed in plants to the south in 2021 and to the north in 2022. The differences may have been caused by the effects of wind direction and speed. We observed wind direction and speed during flowering using Weather Data Acquisition System of Institute for Agro-Environmental Sciences, NARO. Although it was seemed to be some relation between wind direction and outcrossing rate (Supplemental Table 1), it was difficult to determine the relationship between wind direction and crossing rate because of the long flowering period. Further research is needed to determine how flowering timing and wind direction affect crossing rates.

**Fig. 3.**
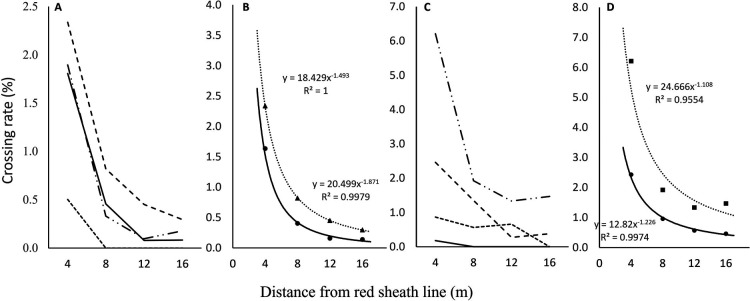
Estimation of pollen dispersal distance of Job’s tears on the basis of crossing rate. A, Crossing rate in each direction in 2021; B, Crossing rate in high-rate direction and average with approximation curve in 2021; C, Crossing rate in each direction in 2022; D, Crossing rate in high-rate direction and average with approximation curve in 2022.

The average crossing rates for areas of four cardinal directions (N, E, S, W) planted with HAT at a 4-m spacing from TUY were 1.6% (4 m), 0.4% (8 m), 0.2% (12 m), and 0.1% (16 m) from the nearest area in 2021, and 2.4% (4 m), 1.0% (8 m), 0.6% (12 m), and 0.5% (16 m) in 2022 (Fig. 3, Supplemental Table 2). To estimate the crossing rates beyond the range of our experiment, we performed a regression analysis. A power regression curve (*y* = 20.499x^–1.871^ *R*^2^ = 0.9979, 2021; *y* = 12.82x^–1.226^ *R*^2^ = 0.9974: 2022) was fitted to the change in the average crossing rate with high probability. The direction with the highest crossing rate was south in 2021 and north in 2022, and the rate change could be estimated by a power regression curve (*y* = 18.429x^–1.493^ *R*^2^ = 1.0, 2021; *y* = 24.666x^–1.108^ *R*^2^ = 0.9554, 2022).

### Estimation of pollen dispersal distance from bordering plants

**Fig. 4.**
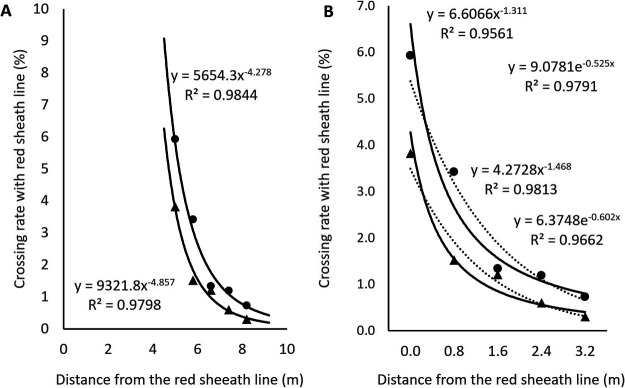
Estimation of pollen intrusion distance into green-sheath Hatoyutaka population on the basis of crossing rate. A, Crossing rates were estimated from the source of red-sheath pollen and are shown with an approximation curve in 2021 (triangles) and 2022 (circles). B, Crossing rates were estimated from the edge of cultivated plants and are shown with a power regression (solid lines) and an exponential equation (dotted lines) in 2021 (triangles) and 2022 (circles), respectively.

To determine the distance that pollen can disperse into a field of plants from bordering plants, we planted a block of HAT plants surrounded by a border of AKI plants, separated by 5 m (Fig. 2C). The rate of crossing with red-sheath plants in the outermost area was 3.8% in 2021 and 5.9% in 2022 (Supplemental Table 3). The crossing rate decreased as the distance from the edge increased, and the decrease rate could be fitted to a power regression curve (*y* = 5654.3x^–4.278^ *R*^2^ = 0.9844, 2021; *y* = 9321.8x^–4.857^ *R*^2^ = 0.9798, 2022; Fig. 4A). However, these decrease rates were larger than those measured in Experiment 2 and did not seem to be good for estimating the crossing rate within 5 m. It is probably the reason that this experimental area includes two different situations, no intervening plants until 5 m and intervening plants after 5 m, and this result may indicate that plants between pollen source and investigated plants reduce the crossing rate. We then estimated the crossing rate from the edge of the cultivated plants and made regression curves. In this situation, two regression curves, a power regression and an exponential equation, were fitted (*y* = 6.6066x^–1.311^ *R*^2^ = 0.9561 and *y* = 9.0781e^–0.525x^ *R*^2^ = 0.9791, 2021; *y* = 4.2728x^–1.468^ *R*^2^ = 0.9813 and *y* = 6.3748e^–0.602x^ *R*^2^ = 0.9662, 2022; Fig. 4B). This result may also indicate the effect of intervening plants on pollen intrusion.

In Experiment 2 in 2022, we set additional areas to the northwest, northeast, southeast, and southwest. The crossing rates were very small (0.0%–0.4%, Supplemental Table 2) compared with the value (0.7%) from the regression equation for this year (*y* = 12.82x^–1.226^) calculated based on the average crossing rate, suggesting that the surrounding HAT plants provide a buffering effect against pollen intrusion.

### Estimation of pollen dispersal rate into a cultivar by unit acreage sampling

In Experiment 3, we estimated that the crossing rate would be less than 1% if plants were harvested 3.2 m from the edge (8.2 m from the pollen source). To validate the estimation and to measure the crossing rate under more natural conditions, we investigated the crossing rate of HAT plants sampled in unit area grown about 5 to 9.8 m from AKI. In this case, we expected to know unevenness of crossing rate in a field that this scenario would reflect the conditions in farmers’ fields. The highest crossing rates were 3.1% and 24.2%, in 2021 and 2022, respectively, in an area 5.0 m distant from AKI. The average crossing rate decreased as the distance increased. However, the crossing rate varied among sampled areas, even within the 5-m harvest area. In addition, some areas that were more than 6 m away from AKI had crossing rates of more than 2.0% (Supplemental Table 4).

## Discussion

### Pollen dispersal

The maximum crossing rate we observed was 36% between adjacent rows (70 cm apart). The outcrossing rate of rice, which is a self-pollinating crop, is less than 0.05% at 0.3 m distance from a pollen donor, although the rate differs by donor size and cultivar (Endo *et al.* 2009); the crossing rate we observed here is much higher. On the other hand, the maximum outcrossing rate between adjoining rows in buckwheat, which is self-incompatible and requires insect pollination (Matsui and Yasui 2020), is approximately 50% with a neighboring plant; the outcrossing rate is sharply reduced within 3 m distance from the pollen source (Adhikari and Campbell 1998). The outcrossing rate in buckwheat does not change markedly between 6 m up to 60 m, indicating that the pollen distribution rule is completely different from that of Job’s tears, probably because of differences in the mode of pollen dispersal.

Maize is a monoecious plant, with male and female flowers formed on separate parts of the same plant; both maize and Job’s tears are in the subfamily Panicoideae. Raynor *et al.* (1972) reported only 0.2% pollen disposition at 60 m distance from the original source. On the basis of the power approximation curve from Experiment 2, we estimated that the pollen disposition of Job’s tears at 60 m would also be about 0.2%, similar to that in maize. Ma *et al.* (2004) reported that the rate of cross-fertilization in maize depended on the distance from the pollen source, the wind direction, and the synchronization of silking and pollen shedding of the two genotypes involved. They also reported an outcrossing rate of up to 82% in the first row adjacent to Bt maize (marker plants); the level of outcrossing was less than 1% beyond the 37th border row (28 m) downwind and the 13th border row (10 m) upwind in all site-years. In addition, their cross-fertilization data with distance to the pollen source were fitted to an exponential equation.

Here, the crossing rate of Job’s tears was also affected by the presence of intervening plants and fitted to an exponential equation when intervening plants were present. However, it was difficult to compare the effect of intervening plants between Job’s tears and corn on the basis of the data, because the experimental designs differed. However, considering the differences in flower position and morphology: in corn, the pistils are usually positioned low on the plant but in Job’s tears the flowers are usually near the top of the plant, the effects of intervening plants on crossing seemed to be weaker in Job’s tears than in corn. Further study may be needed to determine how intervening plants affect pollen intrusion in each species.

### Benefit of the use of red/green sheath as a morphological marker

Pollen dispersal has been investigated in several plant species by using morphological markers, such as dwarfism in buckwheat (Adhikari and Campbell 1998), yellow/white kernel in corn (Kawashima *et al.* 2011, Ma *et al.* 2004), and glutinous endosperm in rice (Endo *et al.* 2009, Tseng *et al.* 2012). Models of pollen dispersal have been created for several crops (e.g., Giddings 2000).

The glutinous/non-glutinous trait is a useful marker that is distinguished by the content of amylose and amylopectin. Because the trait shows a xenia effect it can be distinguished in the crossed seeds. The difference can be recognized by staining with I2-KI: non-glutinous seeds stain purple or dark blue and glutinous seeds stain brown. In the endosperm starch of Jobs’ tears, the amylose content of non-glutinous seeds ranges from 15.9%–25.8% (Li and Corke 1999). On the other hand, glutinous seeds have amylose contents ranging from 0.7%–1.1% (Li and Corke 1999). However, using the glutinous/non-glutinous trait to test pollen dispersal distance is time-consuming and labor-intensive, because the seeds need to be dehulled before they can be stained.

In contrast, although using the sheath color trait requires more time to grow the next generation of plants, it enables us to easily investigate a large number of plants.

### Cultivation of Job’s tears where wild species and non-glutinous endosperm plants are presumed to be grown

As Job’s tears and the wild ancestor can cross in natural environments, it may be necessary to grow them a considerable distance apart in order to avoid crossing. In this study, we estimated that the pollen dispersal distance could be fitted to a power approximation curve or an exponential equation curve, thus allowing us to estimate this distance. However, our other results also showed that crossing rate is not determined by distance alone. In maize, Kawashima *et al.* (2011) reported that the outcrossing rate decreased exponentially but did not reach zero even at 800 m distant; this may also be true for Job’s tears.

Here, we clarified the crossing rate under cultivation conditions and estimated the pollen dispersal distance. Our data could be used as a reference in considering how to cultivate Job’s tears to prevent crossing with wild type or other cultivars. Furthermore, our data could also be used as a guide for planning how to isolate genetically modified plants if they are developed in the future, although we need to consider what may arise after the migration of cultivated seeds in a natural habitat because of the difference of gene flows at the border of the field (Till-Bottraud *et al.* 1992).

## Author Contribution Statement

KM, TT, and AO-T conceived and designed the experiments. KM and KN performed genetic analysis. KM, TT, and AO-T investigated the crossing rates. KM wrote the manuscript, and all authors edited and approved the final manuscript.

## Supplementary Material

Supplemental Figure

Supplemental Tables

## Figures and Tables

**Table 1.** Segregation of red sheath in F_2_ segregating lines

Segregating line	Number of plants (Red: Green)	Expected segregation ratio	χ^2^ value	*P*
Seg_1	79:45	3:1	8.43	*P* < 0.01
		9:7	2.80	0.05 < *P* < 0.10
		13:3	25.40	*P* < 0.01
		15:1	190.97	*P* < 0.01
Seg_2	74:46	3:1	11.37	*P* < 0.01
		9:7	1.43	0.20 < *P* < 0.30
		13:3	30.20	*P* < 0.01
		15:1	210.87	*P* < 0.01
Seg_3	68:47	3:1	15.44	*P* < 0.01
		9:7	0.38	0.50 < *P* < 0.60
		13:3	36.93	*P* < 0.01
		15:1	235.22	*P* < 0.01

**Table 2.** Crossing rates of plants grown in rows spaced at 70 cm (Experiment 1)

Year	Area	Number of plants	Sheath color		Crossing rate (%)
			Red	Green	
2020	A	239	63	176	26.4
	B	262	78	184	29.8
	C	266	39	227	14.7
	D	290	49	241	16.9
2021	A	155	25	130	16.1
	B	189	67	122	35.4
	C	121	35	86	28.9
	D	175	58	117	33.1
