# Supplementary figures and images for: Estimation of pollen dispersal distance in Job’s tears (Coix lacryma-jobi L.) by using red leaf sheath as a morphological marker
Source: Breed Sci. 2023 Sep 9;73(4):408–14. doi: 10.1270/jsbbs.23016 (PMC10722094; doi:10.1270/jsbbs.23016)

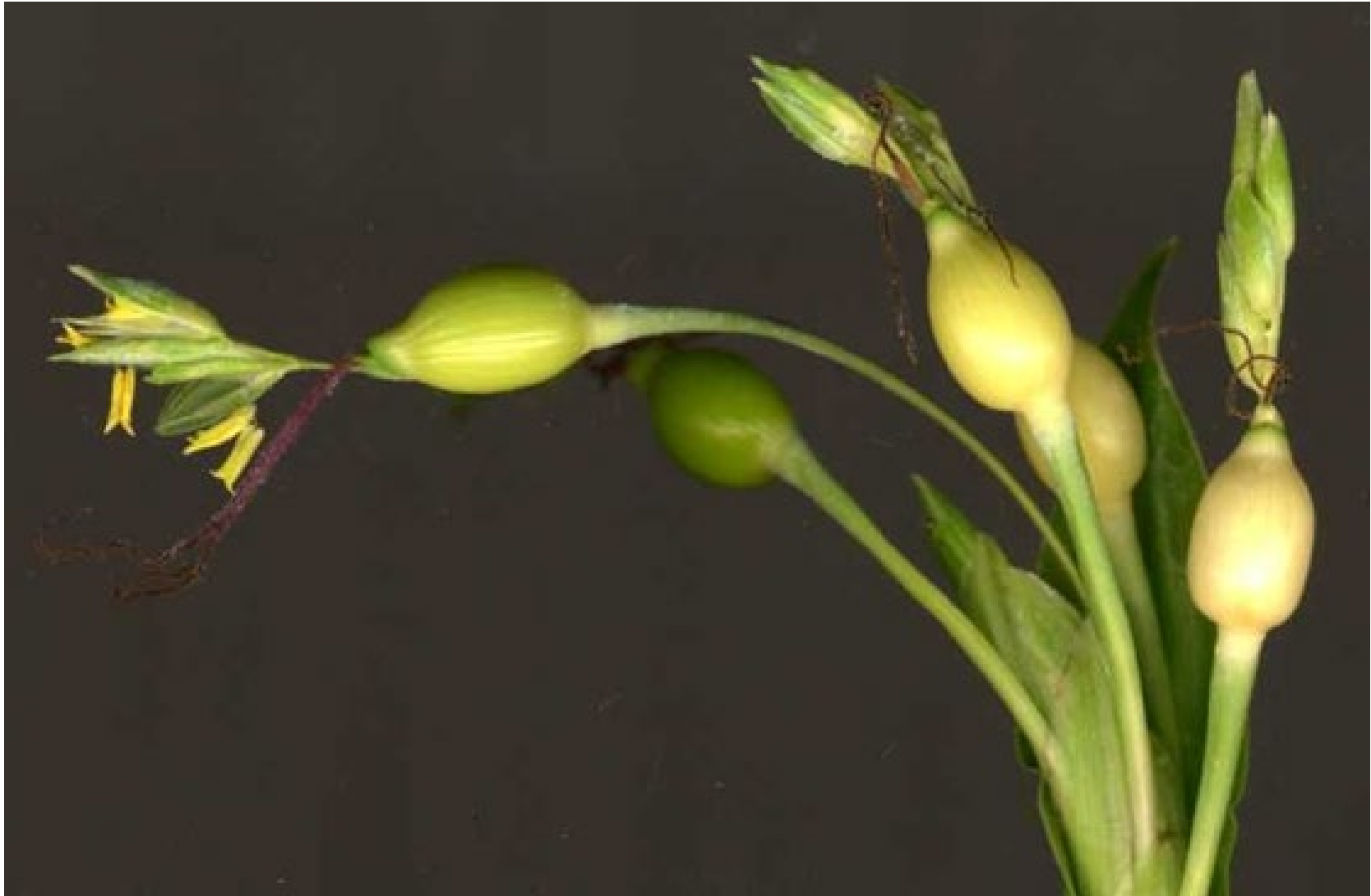

Supplemental Fig. 1 The spikelet of Job's tears

Supplement: Supplementary file 1 — Supplemental Figure [file 73_408_s1.pdf]
