# Supplementary material for: Estimation of pollen dispersal distance in Job’s tears (Coix lacryma-jobi L.) by using red leaf sheath as a morphological marker
Source: Breed Sci. 2023 Sep 9;73(4):408–14. doi: 10.1270/jsbbs.23016 (PMC10722094; doi:10.1270/jsbbs.23016)
Supplement: Supplementary file 2 — Supplemental Tables [file 73_408_s2.pdf]

doi: 10.1270/jsbbs.23016

Supplemental Table 1 The climate information including temperature, wind speeds and wind direction in the Yawara rice field of the NARO.

Year	Month	Templeature (℃)			Wind speed (m/s)		Wind direction	
		Monthly average	Day highest Monthly Average	Day lowest Monthly Average	Monthly average	Maximum	Maximum	Most
2021	July	24.4	28.8	21.2	2.2	9.2	North-NorthWest	East
	August	25.7	30.2	22.1	2.8	12.0	South	South-SouthEast
	September	19.5	23.9	15.5	2.3	7.2	SouthEast	North
2022	July	26.3	31.1	23.1	2.7	7.1	SouthEast	South
	August	26.1	30.8	22.6	2.8	9.7	South-SouthEast	South
	September	22.9	27.5	19.1	2.6	12.0	South	East

The meteorological equipment was located in the Yawara rice field of the NARO in Tsukubamirai, Japan (36º00′24′N, 140º01′14″E, 1 m above sea level) where it was about 1.3 km west of our research fields.

Supplemental Table 2 Crossing rates between pollen-source red-sheath ‘Tsuyakaze’ plants and green-sheath ‘Hatoyutaka’ plants with a 4-m spacing from ‘Tsuyakaze’ plants.

Distance (m)	Direction	Area	2021			2022		
			Red-sheath plants	Total number of plants	Crossing rate (%)	Red-sheath plants	Total number of plants	Crossing rate (%)
4	N	N1	22	1160	1.9	20	322	6.2
	S	S1	29	1241	2.3	8	325	2.5
	E	E1	5	994	0.5	3	347	0.9
	W	W1	21	1163	1.8	1	564	0.2
	Average		19.3	1140	1.6	8.0	389.5	2.4
8	N	N2	4	1206	0.3	7	364	1.9
	S	S2	10	1219	0.8	5	370	1.4
	E	E2	0	1276	0.0	2	353	0.6
	W	W2	6	1311	0.5	0	552	0.0
	Average		5.0	1253	0.4	3.5	409.8	1.0
12	N	N3	1	1027	0.1	5	375	1.3
	S	S3	5	1109	0.5	1	361	0.3
	E	E3	0	1481	0.0	2	303	0.7
	W	W3	1	1251	0.1	0	459	0.0
	Average		1.8	1217	0.2	2.0	374.5	0.6
16	N	N4	2	1120	0.2	5	340	1.5
	S	S4	3	1016	0.3	1	263	0.4
	E	E4	0	1458	0.0	0	291	0.0
	W	W4	1	1211	0.1	0	462	0.0
	Average		1.5	1201	0.1	1.5	339.0	0.5
11.2	NE	NE1	NI	NI	NI	0	185	0.0
	NW	NW1	NI	NI	NI	1	257	0.4
	SE	SE1	NI	NI	NI	0	133	0.0
	SW	SW1	NI	NI	NI	1	276	0.4
	Average					0.5	212.2	0.2
16.8	NE	NE2	NI	NI	NI	0	179	0.0
	NW	NW2	NI	NI	NI	0	231	0.0
	SE	SE2	NI	NI	NI	0	155	0.0
	SW	SW2	NI	NI	NI	0	237	0.0
	Average					0.0	200.5	0.0
100	NW	YA5	4	982	0.4	NI	NI	NI
	SE	YB7	0	1028	0.0	NI	NI	NI
			2.0	1005	0.2			

NI, not investigated

Supplemental Table 3 Estimation of intrusion distance of pollen from red-sheath ‘Akishizuku’ plants into the green-sheath ‘Hatoyutaka’ population, based on crossing rate.

Distance[a] (m)	Plot	2021			2022		
		Red-sheath plants	Total number of plants	Crossing rate (%)	Red-sheath plants	Total number of plants	Crossing rate (%)
5.0	A1	17	445	3.8	23	388	5.9
5.8	A2	5	328	1.5	12	351	3.4
6.6	A3	4	331	1.2	5	373	1.3
7.4	A4	2	332	0.6	5	420	1.2
8.2	A5	1	328	0.3	3	412	0.7

[a] Shortest distance from pollen-source ‘Akishizuku’ (AKI) plants

Supplemental Table 4 Crossing rates between green-sheath 'Hatoyutaka' plants and pollen-source red-sheath 'Akishizuku' plants.

Distance[a] (m)	Plot	2021			2022		
		Red-sheath plants	Total number of plants	Crossing rate (%)	Red-sheath plants	Total number of plants	Crossing rate (%)
5.0	B1-1	6	221	2.7	4	383	1.0
	B1-2	7	225	3.1	7	343	2.0
	B1-3	2	223	0.9	7	327	2.1
	B1-4	2	219	0.9	13	317	4.1
	B1-5	3	218	1.4	93	384	24.2
	Average	4.0	221.2	1.8	24.8	350.8	6.7
5.8	B2-1	1	223	0.5	1	314	0.3
	B2-2	3	207	1.5	5	322	1.6
	B2-3	1	191	0.5	9	339	2.7
	B2-4	0	205	0.0	17	333	5.1
	B2-5	3	222	1.4	31	371	8.4
	Average	1.6	209.6	0.8	12.6	335.8	3.6
6.6	B3-1	1	180	0.6	3	275	1.1
	B3-2	1	190	0.5	0	366	0.0
	B3-3	0	190	0.0	2	390	0.5
	B3-4	4	187	2.1	2	375	0.5
	B3-5	0	182	0.0	7	352	2.0
	Average	1.2	185.8	0.6	2.8	351.6	0.8
7.4	B4-1	2	186	1.1	0	282	0.0
	B4-2	3	181	1.7	2	358	0.6
	B4-3	0	197	0.0	4	379	1.1
	B4-4	5	205	2.4	17	346	4.9
	B4-5	6	145	4.1	3	345	0.9
	Average	3.2	182.8	1.9	5.2	342.0	1.5
8.2	B5-1	0	188	0.0	0	297	0.0
	B5-2	1	156	0.6	7	341	2.1
	B5-3	0	158	0.0	1	358	0.3
	B5-4	0	175	0.0	14	316	4.4
	B5-5	2	200	1.0	2	386	0.5
	Average	0.6	175.4	0.3	4.8	339.6	1.5
9.0	B6-1	0	192	0.0	1	304	0.3
	B6-2	2	187	1.1	3	349	0.9
	B6-3	2	212	0.9	15	341	4.4
	B6-4	0	134	0.0	1	338	0.3
	B6-5	4	177	2.3	3	304	1.0
	Average	1.6	180.4	0.9	4.6	327.2	1.4
9.8	B7-1	0	211	0.0	6	327	1.8
	B7-2	1	200	0.5	0	354	0.0
	B7-3	1	187	0.5	0	305	0.0
	B7-4	2	164	1.2	5	353	1.4
	B7-5	2	177	1.1	1	303	0.3
	Average	1.2	187.8	0.7	2.4	328.4	0.7

[a] Shortest distance from pollen-source 'Akishizuku'(AKI) plants
